# Antigenic Diversity in *Theileria parva* Populations From Sympatric Cattle and African Buffalo Analyzed Using Long Read Sequencing

**DOI:** 10.3389/fgene.2021.684127

**Published:** 2021-07-15

**Authors:** Fiona K. Allan, Siddharth Jayaraman, Edith Paxton, Emmanuel Sindoya, Tito Kibona, Robert Fyumagwa, Furaha Mramba, Stephen J. Torr, Johanneke D. Hemmink, Philip Toye, Tiziana Lembo, Ian Handel, Harriet K. Auty, W. Ivan Morrison, Liam J. Morrison

**Affiliations:** ^1^Royal (Dick) School of Veterinary Studies, Roslin Institute, University of Edinburgh, Edinburgh, United Kingdom; ^2^Ministry of Livestock and Fisheries, Serengeti District Livestock Office, Mugumu, Tanzania; ^3^Nelson Mandela African Institution of Science and Technology, Arusha, Tanzania; ^4^Tanzania Wildlife Research Institute, Arusha, Tanzania; ^5^Vector and Vector-Borne Diseases Research Institute, Tanga, Tanzania; ^6^Liverpool School of Tropical Medicine, Liverpool, United Kingdom; ^7^International Livestock Research Institute, Nairobi, Kenya; ^8^Institute of Biodiversity, Animal Health and Comparative Medicine, College of Medical, Veterinary and Life Sciences, University of Glasgow, Glasgow, United Kingdom

**Keywords:** *Theileria parva*, East Coast fever, African buffalo (*Syncerus caffer)*, molecular epidemiology, cattle

## Abstract

East Coast fever (ECF) in cattle is caused by the Apicomplexan protozoan parasite *Theileria parva*, transmitted by the three-host tick *Rhipicephalus appendiculatus*. The African buffalo (*Syncerus caffer*) is the natural host for *T. parva* but does not suffer disease, whereas ECF is often fatal in cattle. The genetic relationship between *T. parva* populations circulating in cattle and buffalo is poorly understood, and has not been studied in sympatric buffalo and cattle. This study aimed to determine the genetic diversity of *T. parva* populations in cattle and buffalo, in an area where livestock co-exist with buffalo adjacent to the Serengeti National Park, Tanzania. Three *T. parva* antigens (Tp1, Tp4, and Tp16), known to be recognized by CD8^+^ and CD4^+^ T cells in immunized cattle, were used to characterize genetic diversity of *T. parva* in cattle (*n* = 126) and buffalo samples (*n* = 22). Long read (PacBio) sequencing was used to generate full or near-full length allelic sequences. Patterns of diversity were similar across all three antigens, with allelic diversity being significantly greater in buffalo-derived parasites compared to cattle-derived (e.g., for Tp1 median cattle allele count was 9, and 81.5 for buffalo), with very few alleles shared between species (8 of 651 alleles were shared for Tp1). Most alleles were unique to buffalo with a smaller proportion unique to cattle (412 buffalo unique vs. 231 cattle-unique for Tp1). There were indications of population substructuring, with one allelic cluster of Tp1 representing alleles found in both cattle and buffalo (including the TpM reference genome allele), and another containing predominantly only alleles deriving from buffalo. These data illustrate the complex interplay between *T. parva* populations in buffalo and cattle, revealing the significant genetic diversity in the buffalo *T. parva* population, the limited sharing of parasite genotypes between the host species, and highlight that a subpopulation of *T. parva* is maintained by transmission within cattle. The data indicate that fuller understanding of buffalo *T. parva* population dynamics is needed, as only a comprehensive appreciation of the population genetics of *T. parva* populations will enable assessment of buffalo-derived infection risk in cattle, and how this may impact upon control measures such as vaccination.

## Introduction

The protozoan parasite *Theileria parva*, transmitted by the three host tick *Rhipicephalus appendiculatus*, infects cattle and African buffalo (*Syncerus caffer*) across Eastern, Central and Southern Africa (McKeever and Morrison, 1990; Morrison and McKeever, 2006). *T. parva* causes East Coast fever (ECF) in cattle, an acute and often fatal disease, which is responsible for significant economic impact on the livestock industry, estimated at US $596 million annually (GALVmed, 2019). The African buffalo is considered the natural host for *T. parva*, but in contrast to cattle, where mortality can be as high as 90% (Brocklesby, 1961), infected buffalo do not suffer clinical disease. The parasite infects and multiplies within lymphocytes (Baldwin et al., 1988) causing an acute lymphoproliferative disease that can result in death within 3–4 weeks. A proportion of the intra-lymphocytic parasites (schizonts) differentiate to produce merozoites, which upon release, infect erythrocytes giving rise to the tick-infective piroplasm stage. In both host species, a low-level persistent infection, referred to as the carrier state, is established following recovery from the acute phase of infection, facilitating transmission by feeding ticks (Young et al., 1986). The transmission dynamics of *T. parva* between buffalo and cattle are complex (Morrison et al., 2020), with evidence that only a subset of parasites from buffalo are able to differentiate to piroplasms and establish tick-transmissible infections in cattle. However, the nature of this putative population substructuring within *T. parva* is currently unclear.

Cattle have only relatively recently arrived in Africa, having migrated in two broad waves, the first from the fertile crescent approximately 10,000 years ago that established African taurine (*Bos taurus*) breeds, and the second approximately 5,000 years ago involving *Bos indicus* cattle originating from Asia. European *B. taurus* breeds are a more recent introduction into Africa, only arriving in the last 150 years (Hanotte et al., 2002; Kim et al., 2017). This relatively short period of co-existence of cattle with *T. parva* has limited their ability to adapt to the parasite. European *B. taurus* breeds and improved *B. indicus* breeds are highly susceptible, suffering severe disease and high levels of mortality. However, some East African zebu (*B. indicus*) cattle residing in tick-infested areas are more tolerant of *T. parva* infections, with mortality usually <10% in the absence of control measures (Barnett, 1957; Paling et al., 1991). In contrast, *S. caffer* and *T. parva* have co-existed for millennia, allowing co-evolution that has resulted in continued susceptibility to infection but no apparent clinical disease.

Infection of cattle with buffalo-derived *T. parva* results in an acute, usually fatal disease clinically similar to classical ECF (referred to as corridor disease in southern Africa), but with lower levels of parasitized cells in peripheral lymphoid tissues and usually no detectable piroplasms (Young et al., 1977; Sitt et al., 2015). Importantly, such infections are usually not transmissible to ticks (Schreuder et al., 1977). In a few instances, experimental feeding of large numbers of ticks on recovered animals has resulted in transmission and passage of the parasites by ticks in cattle (Barnett and Brocklesby, 1966; Young and Purnell, 1973; Maritim et al., 1992), but it is unclear whether this enables the buffalo *T. parva* parasites to establish in cattle populations in the field.

Population analyses of *T. parva* have demonstrated that in both cattle and buffalo the parasite exists in freely mating (panmictic) populations. However, greater diversity in buffalo-derived *T. parva* has been consistently observed, whether by monoclonal antibody, RFLP, microsatellite or gene sequencing technologies, with analyses indicating that cattle-derived populations comprise a subset of the far greater diversity observed in buffalo (Conrad et al., 1989; Oura et al., 2011b; Hemmink et al., 2018). Additionally, microsatellite and gene sequence analyses of samples from animals naturally infected with *T. parva* have indicated that multiplicity of infection is the norm in both cattle and buffalo, but again buffalo samples having greater diversity, in some instances more than 20 alleles of individual satellite or gene loci detected in single animals (Oura et al., 2011b; Hemmink et al., 2018). This is consistent with the fact that development of immunity does not prevent establishment of infection following subsequent parasite challenge, allowing accumulation of parasite genotypes following repeated parasite challenge.

Experimental studies of immune responses of cattle to *T. parva* infection have provided evidence that immunity is mediated by CD8 T cells specific for parasitized lymphocytes (Morrison and McKeever, 1998). This immunity has been shown to be strain-specific, and the parasite strain-restriction is reflected by strain specificity of CD8 T cell responses (Taracha et al., 1995a, b). Use of parasite-specific CD8 T cell lines for antigen screening has resulted in the identification of a series of CD8 T cell target antigens (Tp1- Tp10) (Graham et al., 2006) and the epitopes within them (Graham et al., 2008) recognized by cells from immune animals. Given the evidence of the importance of CD8 T cell responses in immunity, these antigens have been investigated not only for vaccine development but also to examine antigenic diversity in field populations of *T. parva.*

A study of the sequences of the Tp1 and Tp2 antigen genes in a series of *T. parva*-infected cell lines isolated from cattle or buffalo in different locations in east Africa demonstrated that both of these antigens are highly polymorphic and, by comparing parasites isolated from cattle with those obtained from buffalo or cattle co-grazed with buffalo, the latter were found to contain much greater sequence diversity (Pelle et al., 2011). A further study, utilizing high throughput sequencing of PCR amplicons of 6 *T. parva* antigen genes (Tp1, Tp2, Tp4, Tp5, Tp6, and Tp10), performed on blood samples from buffalo, showed that the genes varied in the extent of polymorphism but confirmed extensive sequence diversity and the presence of multiple alleles in individual animals (Hemmink et al., 2018).

The advent of genomic analysis has added further insight, with the initial sequencing of nine isolates indicating that the two genomes of buffalo-derived *T. parva* analyzed were divergent, with twice the number of single nucleotide polymorphisms (SNPs) compared to the *T. parva* reference genome (*T. parva* Muguga—originally isolated from a cow) compared to seven genomes of cattle-derived *T. parva* (Hayashida et al., 2013). This putative divergence has been recently supported by the first *de novo* genome assembly of a buffalo-derived *T. parva* isolate, which indicated a slightly larger genome, significantly high non-synonymous nucleotide diversity and genome-wide F_*ST*_ values compared to the *T. parva* Muguga genome, at levels compatible with those observed between species rather than within species (Palmateer et al., 2020).

The diversity and transmissibility (to cattle) of buffalo-derived *T. parva* is of obvious relevance with respect to immunity, whether through natural infected tick exposure or via the only currently available vaccine, the “Infection and Treatment Method” (ITM), which involves administration of live *T. parva* (three parasite isolates, including *T. parva* Muguga) and simultaneous treatment with oxytetracycline (Radley et al., 1975a,b,c). This vaccination provides immunity against challenge with cattle *T. parva* (Burridge et al., 1972; Morzaria et al., 1987) which is boosted by challenge. The vaccine has been used successfully to vaccinate cattle in the field, including some areas where buffalo are present (Radley et al., 1979; Radley, 1981; Di Giulio et al., 2009; Martins et al., 2010; Bishop et al., 2015; Sitt et al., 2015), but has been ineffective in protecting cattle introduced into areas of heavy tick challenge grazed only or predominantly by buffalo (Bishop et al., 2015; Sitt et al., 2015). The latter has been interpreted to reflect the greater antigenic diversity in the buffalo-derived *T. parva*.

Areas where buffalo populations interact with cattle are important across sub-Saharan Africa for transmission of multiple infectious diseases (Casey et al., 2014). This is particularly so for *T. parva*, where furthering understanding of the relationship between the parasite populations circulating in cattle and buffalo is a critical factor in resolving both the epidemiology of *T. parva* and vaccine utility. However, few studies have examined *T. parva* genetic diversity in co-circulating populations deriving from sympatric cattle and buffalo. This study aimed to dissect the genetic relationship between *T. parva* identified in cattle and African buffalo sampled in and around the Serengeti National Park, Tanzania, an area endemic for *T. parva*. Long read (PacBio) sequencing was used to obtain sequences from full or near-full length amplicons of genes encoding three antigens recognized by parasite-specific T cells; Tp1, Tp4, and Tp16 (Graham et al., 2006; Tretina et al., 2020; Morrison et al., 2021, submitted), which were selected on the basis of amplifying from *T. parva*, but not from the closely related *T.* sp. buffalo. These data indicated significantly greater allelic diversity in buffalo-derived *T. parva*, with substantial multiplicity of infection in buffalo, and very little sharing of antigen alleles between buffalo and cattle *T. parva* populations. While there was limited evidence for genetic sub-structuring of the two populations, one clade of Tp1 alleles was almost entirely only found in buffalo, suggesting subpopulation host restriction.

## Materials and Methods

### Ethics Statement

Ethical clearance for cattle sampling was gained from the Animal Experimentation Committee of Scotland’s Rural College (SRUC), and the Commission for Science and Technology (COSTECH), Tanzania (Research Permit Number 2016-32-NA-2016-19). Buffalo sampling was carried out with the approval of and in collaboration with the Tanzania Wildlife Research Institute (TAWIRI), as previously described (Casey-Bryars et al., 2018).

### Study Area

**FIGURE 1:**
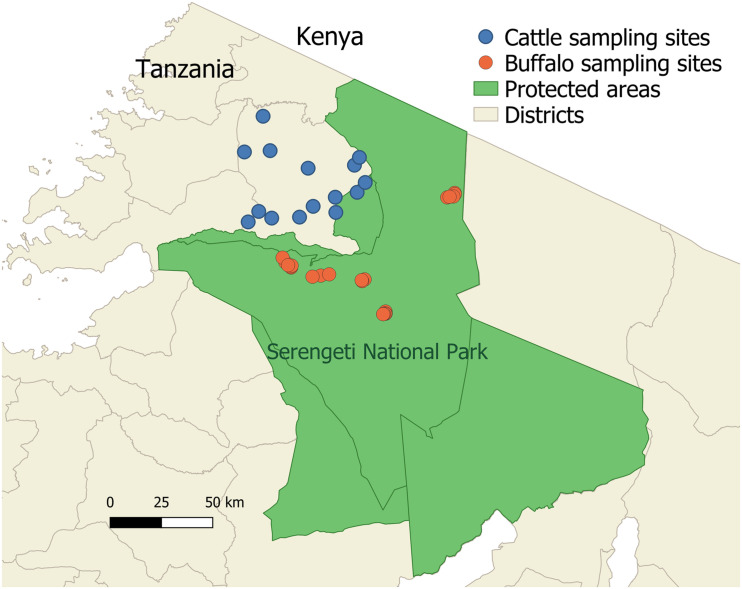
The study area showing locations of cattle and buffalo sampling in the Serengeti-Mara ecosystem. Cattle sampling sites are shown in blue and buffalo sampling sites are shown in orange. Cattle sampling sites (villages) were selected from those close to the protected area boundaries in Serengeti District. Protected wildlife areas are shown in green.

The study was undertaken in the Serengeti National Park, Tanzania and adjacent livestock-keeping areas (Figure 1). The protected areas have unfenced boundaries where livestock can interact with wildlife, including buffalo. Communities in the study area practice livestock keeping as well as mixed crop-livestock farming (Estes et al., 2012). Cattle breeds farmed in this area are predominantly indigenous zebu × Tarime and Zebu × Maswa (Sahiwal, Boran and Mpwapwa zebu cross breeds). Livestock density is highest along the game reserve boundaries north-west and south-west of the National Park (TAWIRI, 2016).

### Sample Collection

Cattle samples were from agropastoral farms, where cattle are herded daily for grazing and water, with herd sizes ranging from 4 to 1,000 cattle. Samples derived from several studies within the study area (Figure 1): In 2016 a randomized cross-sectional survey was designed and carried out in order to gain an estimate of the prevalence of *T. parva* in cattle farmed at the boundary of the Serengeti National Park, using a multistage stratified strategy to select herds, which resulted in 48 herds being sampled (*n* = 770). In 2011 a cross-sectional livestock survey was carried out in a similar study area, which sampled cattle from six herds (*n* = 199). Samples were also analyzed from herds recruited for longitudinal studies on Animal African Trypanosomiasis; samples from four herds were used from 2013, 2015, and 2017 (*n* = 432), selected on the basis of providing resolution for potential analysis of genotypes over space and time. A further subset of the longitudinal cattle samples was used as they derived from cattle described to be clinically ill at the time of sampling (*n* = 201)—while not specifically diagnosed with ECF, these were included in the expectation that some may have been clinical ECF cases (collected in 2013, 2014, and 2015). Buffalo samples (*n* = 22; Figure 1) were collected from the Serengeti National Park in 2011 as previously described (Casey-Bryars et al., 2018). The study groups are referred to as ST1 (cross-sectional survey 2011), ST2 (cross-sectional survey 2016), ST3 (longitudinal 2013), ST5 (longitudinal 2017), ST6 (clinically ill), ST7 (buffalo), and ST8 (reference strain TpM). Sample names used in the manuscript include a designation of study group (ST), village/location of sampling (number) and host (C or B for cattle or buffalo, respectively).

Sampling and handling of cattle was carried out by local Serengeti District livestock officers and a Tanzanian veterinarian. A 10 ml blood sample was collected from the jugular vein into a Paxgene DNA tube (Qiagen). Buffalo samples were collected in the Serengeti National Park by Tanzanian Wildlife Research Institute (TAWIRI) veterinary teams, under strict guidelines regulating wildlife immobilizations. Buffalo were anaesthetized for a short period for jugular venepuncture to collect blood samples, as previously described (Casey-Bryars et al., 2018). Global Positioning System (GPS) location was recorded for all sampled cattle and buffalo (Figure 1).

### DNA Extraction and PCR Amplification

The PAXgene Blood DNA Kit (Qiagen) was used to isolate genomic DNA from whole blood, according to manufacturer’s instructions. DNA was stored at −20 or −80°C for longer term.

#### Diagnostic PCR

A nested p104 PCR (nPCR) was used to screen all field samples for the presence of *T. parva* (Skilton et al., 2002; Odongo et al., 2010). Each PCR reaction (25 μl) consisted of 12.5 μl Quick-Load Taq 2X Master Mix (New England Biolabs), 1 μl of each primer (10 μM), 10 μl nuclease-free water and 1 μl of DNA template. For the second round PCR, first round product was diluted 1:100 in dH_2_O for use as template. The nPCR reactions were carried out in a thermal cycler (MJ Research PTC-200 Engine). First round (primer sequences: 5′ATT TAA GGA ACC TGA CGT GAC TGC 3′ and 5′TAA GAT GCC GAC TAT TAA TGA CAC C 3′) and second round (primer sequences: 5′GGC CAA GGT CTC CTT CAG ATT ACG 3′ and 5′TGG GTG TGT TTC CTC GTC ATC TGC 3′) PCR conditions were as previously described (Skilton et al., 2002; Odongo et al., 2009). The nPCR products were visualized by UV trans-illumination in a 1.5% agarose gel containing GelRed (Biotium) following electrophoresis.

#### Antigen Gene PCR

Samples positive by p104 nPCR were selected for amplification and characterization of *T. parva* antigen genes Tp1, Tp4, and Tp16. Specific primers were designed for nested PCR amplification of full or near full-length gene sequences, with the exception of second round primers for Tp1, previously published by Pelle et al. (2011). Primers were predicted to amplify a 1,618 bp product from Tp1 (TP03_0849: annotated gene length 1,771 bp), 1,473 bp from Tp4 (TP03_0210: 1,740 bp) and 983 bp product from Tp16 (TP01_0726: 1,347 bp). Primer specificity was validated using a panel of DNA (Table 1) from multiple *T. parva* isolates, as well as DNA samples from *T. annulata*, *T. buffeli*, *T. taurotragi*, *T*. sp. (buffalo) and DNA from uninfected *R. appendiculatus* ticks as a negative control. Primers were tested against 22 previously obtained *T. parva* isolates (including cattle and buffalo-derived isolates) to ensure they amplified across the range of strains expected, and were also tested against DNA from 5 *T*. sp. (buffalo) isolates to ensure that primers were specific to *T. parva* and did not amplify product from this closely related species (Bishop et al., 2015; Hemmink et al., 2018), expected to be present in buffalo samples. Each PCR reaction (25 μl) consisted of 5 μl Q5 High-Fidelity Reaction Buffer (New England Biolabs), 0.5 μl dNTPs (Bioline), 1.25 μl of each primer (10μM), 0.25 μl q5 High-Fidelity DNA Polymerase (New England Biolabs), 15.75 μl nuclease-free water and 1 μl of DNA template. For the second round template the first round product was diluted 1:50 in DH_2_O, and a unique barcode sequence was added to the 5′ end of second round primers for each sample (Eurofins Scientific) in order to combine individual amplicons into a single pool, per antigen gene, for multiplexed sequencing^1^. Primer sequences and PCR conditions are shown in Table 2. All PCR reactions were carried out in a MJ Research PTC-200 DNA Engine thermal cycler, and the nPCR products were visualized by UV trans-illumination in a 1.5% agarose gel containing GelRed (Biotium) after electrophoresis. Positive amplicons were subsequently purified using the QIAquick PCR Purification Kit Protocol (Qiagen), according to manufacturer’s instructions.

**TABLE 1:** DNA panel used to test primer specificity.

DNA Sample ID	Country of origin	Sample type
*T. parva* (Muguga)	Kenya	Reference genome
*T. annulata*	Turkey	Non-pathogenic/alternative *Theileria* species
*T. buffeli*	Kenya	
*T. taurotragi*	Kenya	
*T*. sp. (buffalo) 6834 clone 10	Kenya	
*T*. sp. (buffalo) 6998 clone 10	Kenya	
*T*. sp. (buffalo) 6834 clone 5	Kenya	
*T*. sp. (buffalo) 6998 clone 2	Kenya	
*T*. sp. (buffalo) 6998 clone 4	Kenya	
*Rhipicephalus appendiculatus* DNA	Kenya (ILRI)	Negative control
Buffalo clone M3.3	Kenya	Buffalo-derived *T. parva* clones
Buffalo clone M3.6	Kenya	
Buffalo clone M3.7	Kenya	
Buffalo clone M3.9	Kenya	
Buffalo clone M30.2	Kenya	
Buffalo clone M30.5	Kenya	
Buffalo clone M30.8	Kenya	
Buffalo clone M30.11	Kenya	
Buffalo clone M42.2	Kenya	
Buffalo clone M42.5	Kenya	
Buffalo clone M42.8	Kenya	
Buffalo clone M42.12	Kenya	
Buffalo clone 6998.9	Kenya	
Buffalo clone 6998.11	Kenya	
Buffalo-assoc. clone N33.1	Kenya	Buffalo-associated cattle *T. parva* clones
Buffalo-assoc. clone N33.3	Kenya	
Buffalo-assoc. clone N33.4	Kenya	
Buffalo-assoc. clone N33.5	Kenya	
Buffalo-assoc. clone N43.1	Kenya	
Buffalo-assoc. clone N43.3	Kenya	
Buffalo-assoc. clone N43.5	Kenya	
Buffalo-assoc. clone N43.6	Kenya	

**TABLE 2:** Primers and PCR cycling conditions for antigens.

Gene	Primers	Cycling conditions
**Tp1**		
Round 1	F: GCTACGCGGAAATCTAGGCT R: CATCGTTTGCCAGCACTATGA	98°C for 30 s, 40 cycles (98°C for 10 s, 56°C for 20 s, 72°C for 2.5 min), 72°C for 2 min
Round 2*	F: AGGGTCAAAAAAGTTTTATTA R: TTAATTTTTGAGGTAAATTTTG	98°C for 30 s, 37 cycles (98°C for 10 s, 54°C for 20 s, 72°C for 1.5 min), 72°C for 2 min
**Tp4**		
Round 1	F: ATACATCCCAAGGCCAAGCT R: GGAAGGGGTTGGATAGTGCT	98°C for 30 s, 40 cycles (98°C for 10 s, 58°C for 20 s, 72°C for 2.5 min), 72°C for 2 min
Round 2	F: TTACTCATCCTGCCGCTTCT R: TGACCTCCACCTCTCAACAC	98°C for 30 s, 30 cycles (98°C for 10 s, 64°C for 20 s, 72°C for 2 min), 72°C for 2 min
**Tp16**		
Round 1	F: TGATCTACAAGCTCGGTGGA R: GCGGGTATTCTGTGAAGGTC	98°C for 30 s, 35 cycles (98°C for 10 s, 68 C for 20 s, 72°C for 1.5 min), 72°C for 2 min
Round 2	R: AGACATGGGAAAGGGAAGCT F: CCTCCAGTGTCTTTCCGGTA	98°C for 30 s, 32 cycles (98°C for 10 s, 56°C for 20 s, 72°C for 1.5 min), 72°C for 2 min

*Pelle et al. (2011).

### Sample Preparation for Long Read (PacBio) Sequencing

In addition to field samples, amplicons derived from the reference genome stock, *T. parva* Muguga (TpM) were included in each pool as a positive single clone (and allele) control. As a technical control and in order to be able to assess whether the depth of sequencing used captured all diversity present, every sample for Tp1 and Tp4 was submitted for sequencing twice, with a further barcode differentiating these batches (referred to as barcode A or barcode B). Qubit fluorimetry (Thermo Fisher Scientific) was used to calculate equimolar quantities of PCR products for each pool and to verify final DNA concentration of each pool. DNA purity was assessed with NanoDrop spectrophotometry (Thermo Fisher Scientific) and Agilent 2200 TapeStation System (Agilent Technologies) was used to assess DNA integrity and size. Ethanol precipitation was carried out to further purify and concentrate the pooled sample, resulting in 3–5 μg DNA at 50 ng/μl per amplicon sample for library preparation.

The pooled amplicons (96 samples for Tp16; 48 samples for Tp1 and Tp4 due to running A and B replicates) comprised 46 samples that had generated amplicons for Tp1 (34 cattle, 12 buffalo; 3 μg at 61 ng/μl), 47 samples for Tp4 (32 cattle, 15 buffalo; 3.8 μg at 75.4 ng/μl), 94 samples for Tp16 (73 cattle, 21 buffalo; 3.3 μg at 65.6 ng/μl of Tp16), as well as amplicons derived from TpM in each antigen pool (Table 3 for sample details). Each pool was sequenced on a single SMRT cell on a PacBio Sequel machine (Edinburgh Genomics).

**TABLE 3:** Summary of data per sample.

Sample ID	Date sampled	Sex*	Study**	Antigen^†^		
				Tp1	Tp4	Tp16
ST1_01_C01	31/10/2011	F	CS 2011	+	+	-
ST1_01_C02	31/10/2011	F	CS 2011	-	+	-
ST1_01_C03	01/11/2011	F	CS 2011	+	+	-
ST1_01_C04	01/11/2011	F	CS 2011	+	-	-
ST1_02_C05	26/10/2011	F	CS 2011	-	-	+
ST1_02_C06	26/10/2011	M	CS 2011	-	-	+
ST1_02_C07	26/10/2011	F	CS 2011	-	-	+
ST1_02_C08	26/10/2011	F	CS 2011	-	-	+
ST1_02_C09	27/10/2011	M	CS 2011	-	-	+
ST1_02_C10	27/10/2011	M	CS 2011	-	-	+
ST1_02_C11	27/10/2011	F	CS 2011	+	+	+
ST1_03_C12	01/08/2011	F	CS 2011	+	-	+
ST1_04_C13	27/07/2011	F	CS 2011	-	-	+
ST1_04_C14	27/07/2011	F	CS 2011	+	-	+
ST1_04_C15	27/07/2011	F	CS 2011	-	-	+
ST1_04_C16	27/07/2011	F	CS 2011	-	+	+
ST1_04_C17	27/07/2011	F	CS 2011	+	-	+
ST1_05_C18	24/10/2011	M	CS 2011	+	-	+
ST1_05_C19	24/10/2011	M	CS 2011	+	-	+
ST1_05_C20	24/10/2011	M	CS 2011	-	-	+
ST1_05_C21	24/10/2011	M	CS 2011	+	+	+
ST1_05_C22	25/10/2011	M	CS 2011	-	-	+
ST1_05_C23	25/10/2011	M	CS 2011	-	-	+
ST1_05_C24	25/10/2011	M	CS 2011	+	+	+
ST1_06_C25	25/07/2011	F	CS 2011	-	-	+
ST1_06_C26	25/07/2011	F	CS 2011	-	+	+
ST1_06_C27	25/07/2011	M	CS 2011	-	-	+
ST1_06_C28	25/07/2011	F	CS 2011	-	-	+
ST1_06_C29	25/07/2011	F	CS 2011	-	-	+
ST1_06_C30	25/07/2011	F	CS 2011	-	-	+
ST1_06_C31	25/07/2011	F	CS 2011	-	-	+
ST1_06_C32	25/07/2011	F	CS 2011	-	-	+
ST1_06_C33	26/07/2011	M	CS 2011	-	-	+
ST1_06_C34	26/07/2011	F	CS 2011	+	-	+
ST1_06_C35	26/07/2011	F	CS 2011	-	-	+
ST1_06_C36	26/07/2011	F	CS 2011	-	-	+
ST2_01_C37	21/07/2016	F	CS 2016	-	+	+
ST2_02_C38	18/07/2016	M	CS 2016	+	+	+
ST2_02_C39	18/07/2016	F	CS 2016	+	+	+
ST2_02_C40	20/07/2016	F	CS 2016	+	+	+
ST2_02_C41	19/07/2016	F	CS 2016	-	-	+
ST2_02_C42	19/07/2016	F	CS 2016	+	+	+
ST2_02_C43	19/07/2016	M	CS 2016	+	+	+
ST2_03_C44	04/08/2016	M	CS 2016	-	-	+
ST2_03_C45	04/08/2016	F	CS 2016	+	+	+
ST2_03_C46	05/08/2016	F	CS 2016	-	-	+
ST2_03_C47	05/08/2016	F	CS 2016	+	+	+
ST2_03_C48	05/08/2016	F	CS 2016	-	-	+
ST2_04_C49	10/08/2016	F	CS 2016	-	-	+
ST2_04_C50	11/08/2016	M	CS 2016	-	+	+
ST2_04_C51	11/08/2016	F	CS 2016	+	+	+
ST2_04_C52	11/08/2016	F	CS 2016	-	-	+
ST2_05_C53	28/07/2016	F	CS 2016	+	+	+
ST2_05_C54	28/07/2016	M	CS 2016	-	-	+
ST2_06_C55	08/08/2016	F	CS 2016	-	+	+
ST2_06_C56	08/08/2016	F	CS 2016	+	+	+
ST2_06_C57	09/08/2016	M	CS 2016	+	-	+
ST2_06_C58	09/08/2016	F	CS 2016	+	-	+
ST2_06_C59	09/08/2016	M	CS 2016	-	+	+
ST2_07_C60	25/07/2016	F	CS 2016	+	-	+
ST2_07_C61	25/07/2016	F	CS 2016	+	-	+
ST3_01_C62	15/04/2013	F	Long 2013	+	+	+
ST3_02_C63	15/05/2013	F	Long 2013	-	-	+
ST3_02_C64	15/05/2013	M	Long 2013	-	-	+
ST3_02_C65	15/05/2013	F	Long 2013	+	-	+
ST3_02_C66	15/05/2013	M	Long 2013	-	-	+
ST4_01_C67	20/01/2015	?	Long 2015	-	+	+
ST4_01_C68	28/01/2015	M	Long 2015	-	-	+
ST4_02_C69	14/03/2015	F	Long 2015	-	-	+
ST4_02_C70	14/03/2015	F	Long 2015	-	-	+
ST5_01_C71	18/05/2017	F	Long 2017	+	+	+
ST5_01_C72	18/05/2017	F	Long 2017	+	+	+
ST5_02_C73	30/05/2017	M	Long 2017	+	+	+
ST5_02_C74	30/05/2017	M	Long 2017	-	-	+
ST5_02_C75	30/05/2017	F	Long 2017	-	+	-
ST5_03_C76	10/05/2017	F	Long 2017	+	+	+
ST6_01_C77	09/09/2014	F	Sick 2014	+	+	+
ST6_01_C78	09/09/2014	F	Sick 2014	+	+	-
ST7_01_B01	01/07/2011	F	Buffalo 2011	-	-	+
ST7_01_B02	01/07/2011	F	Buffalo 2011	+	+	+
ST7_01_B03	01/07/2011	M	Buffalo 2011	+	+	+
ST7_01_B04	01/07/2011	F	Buffalo 2011	-	+	+
ST7_01_B05	01/07/2011	F	Buffalo 2011	-	+	+
ST7_01_B06	02/07/2011	M	Buffalo 2011	+	+	+
ST7_01_B07	02/07/2011	M	Buffalo 2011	-	+	+
ST7_01_B08	02/07/2011	M	Buffalo 2011	-	+	+
ST7_01_B09	02/07/2011	M	Buffalo 2011	+	+	+
ST7_01_B10	02/07/2011	M	Buffalo 2011	+	+	+
ST7_01_B11	03/07/2011	M	Buffalo 2011		+	+
ST7_01_B12	03/07/2011	F	Buffalo 2011	+	+	+
ST7_01_B13	03/07/2011	F	Buffalo 2011	-	-	+
ST7_01_B14	05/07/2011	?	Buffalo 2011	-	-	+
ST7_01_B15	05/07/2011	M	Buffalo 2011	+	+	+
ST7_01_B16	05/07/2011	M	Buffalo 2011	+	-	+
ST7_01_B17	06/07/2011	M	Buffalo 2011	+	+	+
ST7_01_B18	06/07/2011	M	Buffalo 2011	+	-	+
ST7_01_B19	06/07/2011	F	Buffalo 2011	-	+	+
ST7_01_B20	06/07/2011	F	Buffalo 2011	+	-	+
ST7_01_B21	06/07/2011	F	Buffalo 2011	+	+	+

*M = Male; F = Female; ? = sex unrecorded. **Study groups are: CS 2011 (cross-sectional survey 2011; ST1), CS 2016 (cross-sectional survey 2016; ST2), Long 2013 (longitudinal 2013; ST3), Long 2015 (longitudinal 2015; ST4), Long 2017 (longitudinal 2017; ST5), Sick 2014 (clinically ill; ST6) and Buffalo 2011 (buffalo; ST7). ^†^Symbols represent successful (+) or unsuccessful (-) amplification of antigen gene by PCR.

### PacBio Sequencing Analysis

#### Raw Data Processing

PacBio raw unfiltered multiplexed sub-reads were received in bam format from Edinburgh Genomics for each of the three separate PacBio runs containing reads from each antigen—Tp1, Tp4, and Tp16, respectively. PacBio circular consensus caller (PBCCS v3^2^) was used to generate CCS reads from sub-reads. PacBio barcode demultiplexer (lima v1^3^) was used to demultiplex the CCS reads into their respective biological replicate samples using the primer barcode information used in the PCR amplification step. PacBio long read aligner (Blasr^4^) was used to align CCS reads from each sample to their respective reference amplicon sequence and outputted in the machine parsable format using the –placeGapConsistently option for better variant calling downstream using downstream data analysis scripts. Data and scripts are available via the following link: https://doi.org/10.7488/ds/3003. The main script files for each data set are titled Tp1_Variant_Analysis.m, Tp4_Variant_Analysis.m and Tp16_Variant_Analysis.m, respectively (please note that within the data files Tp16 is referred to by the alias N60).

#### Read QC and Filtering

CCS reads were filtered to retain only high-quality reads for variant calling and allelic distribution using the following criteria (PCR replicates accounted as separate samples at this stage); (a) sequenced length of a CCS read length should fall within the mean ± SD sequence length distribution window for reads coming from all samples of a given antigen, to remove short fragments and long artifacts, (b) using the PCR primers as a guide, each CCS read was tested to make sure they were full length amplicons and remove all fragmented reads, (c) the PacBio read quality score was used to retain only high confidence reads with score RQ > 0.99 from the consensus caller, (d) for Tp1 and Tp16, genomic amplicons without introns translated in frame and reads with non-sense codons in open reading frame (ORF) were filtered out.

#### Variant Calling and Allelic Distribution

**FIGURE 2:**
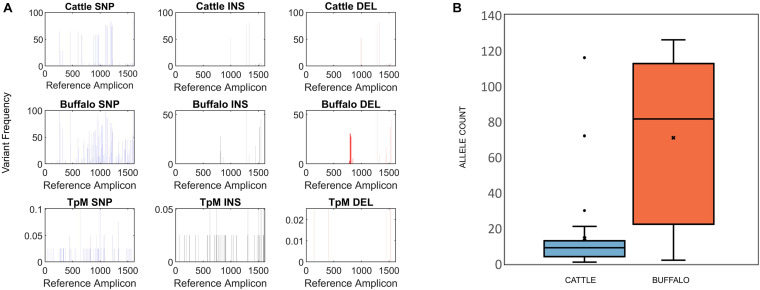
**(A)** Single Nucleotide Polymorphism (SNP), insertion (INS) and deletion (DEL) frequency for amplicon data across the length of the Tp1 amplicon, with respect to the TpM reference sequence. The mean calculated SNP/INDEL frequency is shown at each base location for the Tp1 gene. Blue bars represent SNPs, black bars represent insertions and red bars represent deletions. **(B)** Tp1 allele counts in cattle- (blue) and buffalo-derived (orange) samples. Median (line), mean (x) and standard deviation (whiskers) are shown, dots represent outlier samples with extreme allele counts.

CCS reads passing the above filters were analyzed for their Blasr alignment to their respective reference amplicon and variant calling was done using an in-house pipeline^5^. The CCS reads were grouped based on whether the reads originated from cattle, buffalo or TpM samples. For each of the groups, variant frequency at each base pair locus was calculated for SNPs and insertions/deletions (INDELs) based on their alignments against the reference sequence (Figure 2A).

SNP and INDEL sites identified were filtered for variant frequency greater than 1% for Tp1 and Tp16 samples and greater than 5% for Tp4 samples to account for background error introduced by PacBio sequencing, such as homopolymer errors, for each of the source group—cattle, buffalo and TpM—samples. For each source group, the SNP/INDEL which passed the variant frequency threshold from each sequenced read was substituted into the reference amplicon sequence, in order to remove any background noise from the sequenced reads. For Tp1 and Tp16 an additional filtering step was applied to the modified reads to remove any alleles which failed to produce a complete ORF, by removing any alleles which were out of frame or introduced non-sense mutation during SNP/INDEL substitution. Unique alleles were identified by clustering the modified reads originating from cattle and buffalo at 100% identity. The sequenced read count evidence supporting each identified allele was summarized across the total sequenced pool (cattle, buffalo, TpM) and each individual sample replicate (Supplementary Data 1–3). For Tp1 and Tp16, each allele was tested against the reference to check if the mutation was synonymous or non-synonymous. For Tp1, the known epitope region (Nanteza et al., 2020) was selected from each allele sequence to list all unique epitope residues. Ts/Tv ratio was calculated for each source group Tp1 samples—cattle, buffalo, TpM. Identified alleles were summarized for each animal (replicates combined) into total alleles, alleles shared between two replicates, and alleles in replicate A and replicate B separately. To assess the allelic diversity and potential genetic relationships between cattle- and buffalo-derived *T. parva* populations, a phylogenetic (neighbor-joining) tree was constructed using MEGA v.7.

## Results

### Detection of *Theileria parva* in Field Samples and Amplification of Antigen Genes

A nested p104 PCR assay was used to screen 1,602 cattle and 22 buffalo samples, of which 126 cattle (7.87%) and 22 buffalo (100%) were positive for *T. parva*. Primers designed to amplify full or near-full length gene sequences of the antigens Tp1, Tp4, and Tp16 were applied to the 126 cattle and 22 buffalo *T. parva* positive samples. A PCR product of the predicted size (1,618 bp) was obtained from 34 cattle and 12 buffalo samples for Tp1, 32 cattle samples and 15 buffalo samples for Tp4 (1,473 bp), and 80 cattle and 21 buffalo samples for Tp16 (983 bp). All 46 samples for Tp1 (34 cattle, 12 buffalo) and 47 samples for Tp4 (32 cattle, 15 buffalo) that generated PCR products, as well as amplicons deriving from TpM, were submitted in duplicate for sequencing (Table 3). For Tp16, 94 samples that had generated amplicons (73 randomly selected cattle and 21 buffalo), along with TpM amplicons, were submitted for sequencing (no duplication; Table 3).

### Tp1 PacBio Sequencing

Using PacBio long read sequencing, a total of 404,723 raw CCS reads was obtained; 282,998 for cattle-derived samples (34 samples, submitted in duplicate), 113,149 for buffalo-derived samples (12 samples, submitted in duplicate) and 8,576 for TpM (*n* = 2; Supplementary Data 1).

After filtering steps based upon read quality (RQ ≥ 0.99; 356,440 reads passed this threshold; 250,139 for cattle, 98,710 for buffalo and 7,591 for TpM), read length (mean ± 1 SD; 311,618 full length reads; 222,726 for cattle, 82,130 for buffalo, and 6,762 for TpM), codon length (checking in-frame; 224,513 reads; 166,801 for cattle, 52,517 for buffalo, and 5,195 for TpM) and ORF (checking for stop codons; 160,326 reads; 128,200 for cattle, 28,013 for buffalo, and 4,113 for TpM), there was a total of 155,159 reads remaining (38.3% of starting total); 125,851 from 68 cattle samples (average of 1,850 reads per sample), 25,359 for 24 buffalo samples (average of 1,056 reads per sample), and 3,949 for TpM (average of 1,974 per TpM sample).

### Tp1 Alleles

At the SNP/INDEL frequency threshold of 1%, mismatches, deletions and insertions were identified at 110, 48 and 13 nucleotide positions, respectively, i.e., there were a total of 171 variable nucleotide positions across the 1,618 bp amplicon (Figure 2A). This resulted in a total of 3,876 alleles being initially identified. However, a filter was applied to avoid potential errors deriving from single reads (i.e., to be considered in our dataset a sequence must be represented by two independent reads), which resulted in a final allele count of 651, which included the reference TpM allele. The read count of this final filtered dataset was 150,198.

Importantly, the data filtering steps resulted in only a single allele being detected in the TpM control dataset (3,949 reads for TpM; 2,165 reads in replicate A and 1,784 reads in replicate B), consistent with expectations as this DNA derived from a clonal *in vitro* cultured *T. parva* cell line, and suggesting filtering steps were appropriately stringent. Notably, the TpM sequence was also identified in one buffalo (294 reads) and eight cattle (7,715 reads).

Buffalo samples on average had much higher allelic diversity than cattle, with a median of 81.5 alleles (S.D. = 45.2) being identified in buffalo compared to a median of nine alleles (S.D. = 21.8) in cattle samples (Figure 2B). Buffalo samples ranged from 126 alleles identified in ST7_01_B06 to 22 alleles in ST7_01_B02, with two buffalo samples, ST7_01_B18 and ST7_01_B20, having relatively low numbers of alleles detected, 10 and 2, respectively. Cattle sample-derived allele numbers ranged from 30 in ST1_04_C14 to a single allele in ST2_04_C51, with the exception of samples ST1_05_C21 and ST2_03_C45, in which 116 and 72 were detected, respectively. After data filtering, cattle sample ST1_01_C03 had no detectable alleles, and so this sample was removed from further analysis. The number of alleles identified did not necessarily correlate directly with number of reads—for example, for the cattle-derived samples ST1_05_C21 and ST2_03_C45, which were outliers in terms of high numbers of alleles (116 and 72), there were 3,281 and 6,067 reads, compared to the other cattle from same study groups; 1,443 (ST1_05_C18—2 alleles), 1,720 (ST1_05_C19—9 alleles), 4,400 reads (ST1_24_C24—13 alleles) and 6,785 reads (ST3_05_C47—3 alleles). Similarly, the buffalo-derived samples ST7_01_B18 and ST7_01_B20 which had unusually low allele counts (10 and 2, respectively) did not have significantly lower read counts compared to other samples in the buffalo-derived sample cohort. These data suggest that it is not simply differential sequencing coverage that is responsible for the high or low allele detection in these samples.

**FIGURE 3:**
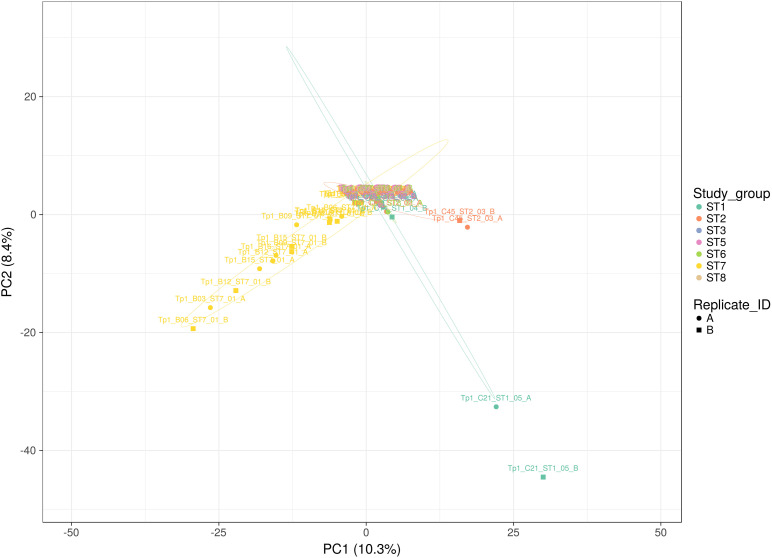
Principal Component analysis applied to Tp1 alleles present in all individual replicates (A or B representing paired independent sequencing data from the same sample). Study group ST1 (aqua) represents cross-sectional cattle from 2011, ST2 (pink) represents cross-sectional cattle from 2016, ST3 (blue) represents longitudinal cattle from 2013, ST5 (lilac) represents longitudinal cattle from 2017, ST6 (green) represents clinically ill cattle, ST7 (yellow) represents buffalo, and ST8 (beige) represents reference strain TpM. Replicates A and B are represented by a circle and a square respectively. The proportion of variation in the dataset, explained by the 1st and 2nd principal components, is indicated in parenthesis on each axis.

Each individual sample was sequenced twice independently, resulting in two replicate datasets (A and B) from the same amplicon (note that this pertains to 36 samples; for samples ST7_01_B21, ST7_01_B20, ST5_01_C71, ST1_01_C03, ST7_01_B02, ST6_01_C78, ST2_05_C53, ST2_06_C57, ST2_04_C51 and ST1_01_C03 data was only successfully generated for one replicate). This approach provided the ability to assess the coverage of sequencing with respect to allele detection (summarized in Figure 3). The alleles detected in buffalo-derived replicates A and B for the same samples, while showing a degree of overlap were very often different, indicating that the depth of sequencing coverage was not sufficient to detect all alleles in a sample. In contrast, sequencing replicates of cattle-derived samples indicated much greater sharing of alleles, with the exception of outlying cattle C21 and C45 (Figure 3) between the replicates of individual samples, suggesting that for cattle-derived samples the sequencing coverage was sufficient to identify all or most alleles present. TpM replicates were identical, as expected with a single allele control. These data confirm that the diversity in buffalo-derived samples is much greater than in cattle-derived samples, and indeed indicate that the approach taken was not sufficient to identify all alleles potentially present in buffalo-derived samples.

### Tp1 Allele Sharing

**FIGURE 4:**
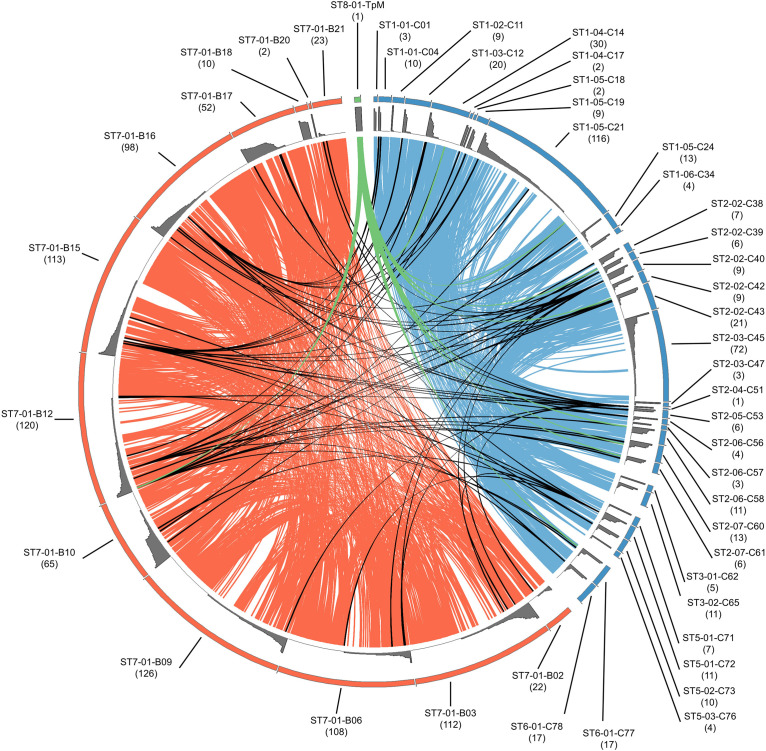
Relationship of alleles within and between buffalo- and cattle-derived samples. Individual samples are arranged around the outer ring, as indicated by labels with sample name (blue indicating cattle and orange indicating buffalo); the size of the segment in the outer ring corresponds with the allele count per individual (also noted in brackets under sample name), with the relative abundance of each allele illustrated by the gray histogram. Alleles shared between buffalo samples are joined by orange ribbons, alleles shared between cattle samples are joined by blue ribbons, and alleles shared between buffalo and cattle are joined by black ribbons. TpM is shown in green, and is joined to animals in which TpM was detected by a green ribbon.

When allele sharing within and between species groups was analyzed, sharing clearly commonly occurs within cattle or within buffalo (blue or orange ribbons, respectively, in Figure 4), whereas sharing between species (black ribbons in Figure 4) was much less frequent. Of the 420 alleles detected in buffalo, 412 alleles were unique to buffalo and not detected in cattle samples. 239 alleles were identified in cattle samples, and of those 231 alleles were only found in cattle samples. Therefore, only eight of the 651 alleles were present in both cattle and buffalo, and this included the reference TpM allele (green ribbon in Figure 4). Most of the cattle-unique alleles derive from the two samples with high allele counts (ST1_05_C21 and ST2_03_C45), with 118/231 cattle-derived alleles (51.1%) being detected only in these samples.

Of the eight alleles shared between species (Figure 4), four alleles were found in multiple buffalo and a single cow (alleles #0304, #0461, #0501, and #0300). One allele was found in one buffalo and one cow (#0120), and two alleles (#0055 and TpM) were found in multiple cattle and a single buffalo, TpM being present in eight and #0055 in seven cattle, respectively. It is difficult to discern patterns with such low numbers of shared alleles.

Of the 34 cattle, 12 did not share any alleles with buffalo (ST1_01_C01, ST1_02_C11, ST1_05_C18, ST1_06_C34, ST2_02_C43, ST2_03_C45, ST2_03_C47, ST2_06_C57, ST3_01_C62, ST3_02_C65, ST5_01_C71, ST5_03_C76; Figure 4). For the two cattle samples with unusually high allele numbers, cow ST2_03_C45 (72 alleles) shared no alleles with buffalo and ST1_05_C21 (116 alleles) shared four alleles with four buffalo (B10, B12, B15, B16), including the TpM allele. For the 12 buffalo samples that gave data, only two buffalo, the samples with low allele counts (ST7-01-B20 and ST7-01-B18), shared no alleles at all with cattle. The buffalo samples with the highest allele counts, B12 (120), B15 (113), and B16 (98) each had four alleles shared with cattle.

These data further indicate that for cattle and buffalo the degree of sharing between species was low, although for buffalo most animals had at least some alleles that are detected in cattle, whereas for cattle (where the data indicate that we have the resolution to detect all alleles in the samples), a substantial proportion did not have any alleles that are also present in buffalo.

Clearly most alleles detected were unique to each species in the dataset. Of the 231 cattle-unique alleles, 90.9% were shared between two or more cattle, and this ranged from being shared between two animals to being shared between 19 animals. For example, 93 alleles (40.3%) were present in two cattle, 43 alleles (18.6%) in three, 22 alleles (9.5%) in four, 12 alleles (5.2%) in five cattle, and 8 alleles (3.5%) in six cattle. The remaining 32 alleles were shared between seven and 19 cattle; of the most frequently occurring, allele #0002 was present in 19 cattle (read counts ranging from 1 to 6,781), allele #0021 was in 17 cattle (1–6,867) and allele #0031 was in 10 cattle (1-2). These data indicate that there were frequently shared alleles between cattle, but the most frequently shared were not necessarily the most abundant allele in all cattle.

Of the 412 buffalo-unique alleles, almost all were shared across at least two animals, with only three alleles being found in just one animal. 220 (53.4%) alleles were present in two buffalo, 90 alleles (21.8%) in 3 buffalo, 46 (11.2%) in four buffalo, 25 (6.1%) in five buffalo, and 18 (4.4%) in six buffalo. The remaining five most frequently encountered alleles were found in seven (alleles #611 and #771; read counts ranging from 1 to 4 and 5 to 524, respectively), eight (alleles #601 and #667; read counts 1–3 and 21–378, respectively) and ten buffalo (allele #0590; read counts ranging from 1 to 247). Again, these data indicate that there is not a correlation between the degree of sharing and allele dominance in individual buffalo.

### Tp1 Allele Phylogeny

**FIGURE 5:**
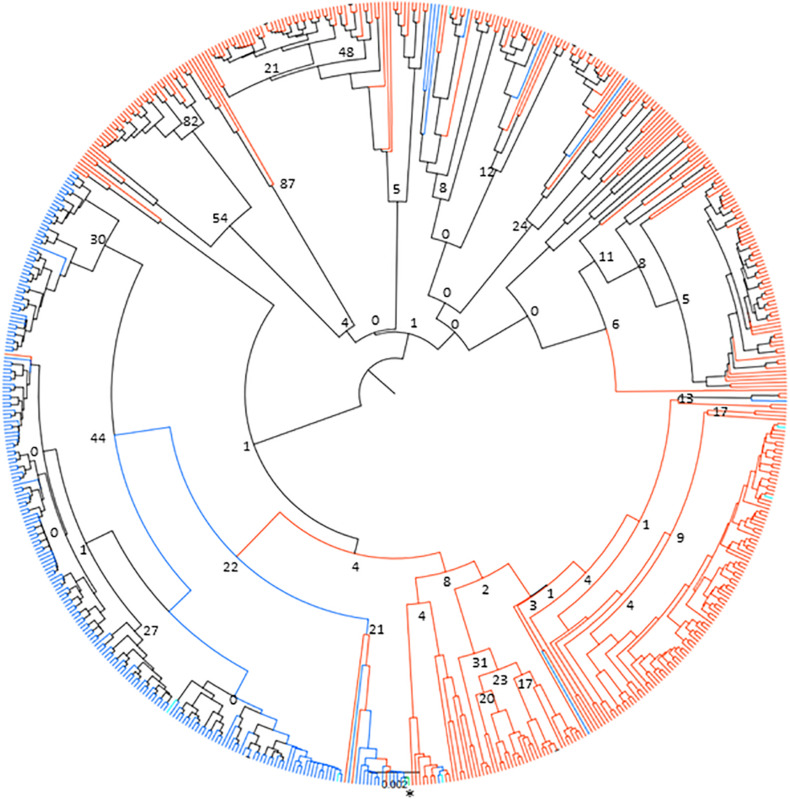
Neighbor-joining phylogenetic tree of 651 Tp1 alleles found in cattle and buffalo samples. Alleles only found in cattle are shown in blue, alleles only found in buffalo are shown in orange and alleles present in both cattle and buffalo are shown in turquoise. The TpM reference sequence allele is highlighted in green and marked with an asterisk. Bootstrap values are shown at selected tree nodes.

The lack of sharing between species could be due to many reasons, including potential genetic substructuring reflecting either adaptation to the respective host species or different transmission cycles. In order to investigate potential substructuring by host species, the genetic relationship between alleles found in cattle and buffalo was analyzed using a neighbor-joining clustering approach. The data indicated that there were two main clusters of alleles, one mainly buffalo-derived cluster, and one that broadly split into cattle-derived alleles and buffalo-derived alleles (Figure 5), with TpM being in the mainly cattle-derived sub-cluster. There were only six cattle alleles, and one allele found in both species, that group with the main buffalo-derived cluster, with all other alleles found in cattle or both species being present in the second cattle/buffalo cluster. While the bootstrap values indicated little support overall for strong signals of substructuring (perhaps not surprising given the relatively few variable sites across the dataset), and the small number of alleles shared between species limited what can be concluded, the species-specific clustering of alleles was very marked.

### Tp1 CTL Epitopes

A Tp1 epitope recognized by the bovine immune response has been previously described (Graham et al., 2006, 2008; MacHugh et al., 2009; Pelle et al., 2011). This epitope sequence was analyzed to understand the diversity of a sequence that has been defined as interacting with the host immune response, to examine if there was any relationship between epitope identity and host species. Across the 651 Tp1 alleles, five epitope variants (i.e., nucleotide sequences that result in an amino acid change) were identified in the previously described sequence (VGYPKVKEEML in TpM) that demarcates an epitope recognized by protective cytotoxic CD8 T cells (Table 4). The predominant variant circulating in cattle was VGYPKVKEEII, found in 31 cattle (73,202 reads), followed by VGYPKVKEEML in 24 cattle (50,791 reads), VGYPKVKEEMI in 12 cattle (196 reads) and VGYPKVKEEIL in six cattle (56 reads). In buffalo, variant VGYPKVKEEML was predominant, found in all 12 buffalo (19,896 reads), followed by VGYPKVKEEII in nine buffalo (1,805 reads), VGYPKVKEEMV in four buffalo (301 reads) and VGYPKVKEEIL in a single buffalo (2 reads). The variant with MI at positions 10 and 11 was therefore only found in cattle, and that with MV was only found in buffalo.

**TABLE 4:** Tp1 epitope variants found in cattle and buffalo samples.

Epitope	Number cattle	Cattle reads	Number buffalo	Buffalo reads
VGYPKVKEEML	24	50,791	12	19,896
VGYPKVKEEMV	0	0	4	301
VGYPKVKEEMI	12	196	0	0
VGYPKVKEEIL	6	56	1	2
VGYPKVKEEII	31	73,202	9	1,805

### Tp4 and Tp16 Alleles

**FIGURE 6:**
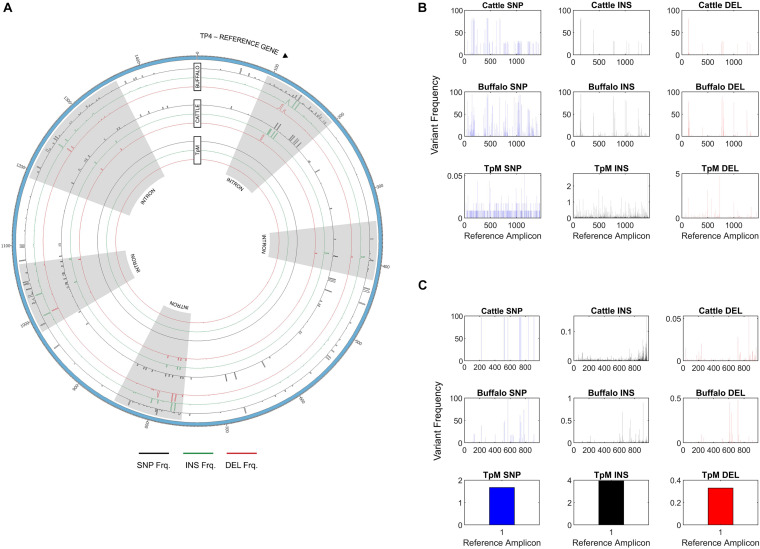
**(A)** Single Nucleotide Polymorphism (SNP), insertion and deletion (INS/DEL) frequencies across the amplicon length of Tp4, with respect to the TpM reference sequence, highlighting increased mutation rates in intronic regions. SNPs are represented in black, insertions in green, and deletions in red. The outermost histogram represents buffalo, the middle represents cattle and the inner represents TpM. Intronic regions are highlighted in gray. **(B)** SNP/INDEL frequency for amplicon data across the length of the Tp4 amplicon, with respect to the TpM reference sequence. The mean calculated SNP/INDEL frequency is shown at each base location for the Tp4 gene. Blue bars represent SNPs, black bars represent insertions and red bars represent deletions. **(C)** SNP/INDEL frequency across the length of the Tp16 amplicon, with respect to the TpM reference sequence. The mean calculated SNP/INDEL frequency is shown at each base location for the Tp16 gene. Blue bars represent SNPs, black bars represent insertions and red bars represent deletions.

**FIGURE 7:**
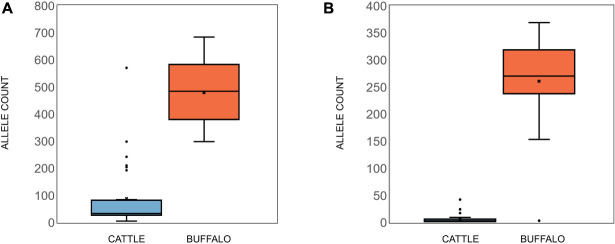
**(A)** Tp4 and **(B)** Tp16 allele counts in cattle (blue) and buffalo (orange). Median (line), mean (x), and standard deviation (whiskers) are shown.

In addition to Tp1, analysis of two additional antigens recognized by CD8 or CD4 T cells, Tp4 and Tp16, was also carried out (Supplementary Data 2 and 3). For Tp4, amplicons from 48 samples (32 cattle, 15 buffalo and TpM) were sequenced in duplicate (total *n* = 96). A total of 74,274 alleles were initially identified, but after the removal of intronic regions (Figure 6A) and filtering (a sequence must be represented by two independent reads), the final allele count was 4,449 (286,100 reads). A single TpM allele was identified (11,422 reads) at the SNP/INDEL variant frequency threshold of 5% used (Figure 6B). The median allele count in cattle samples was 32.5 (SD = 116.4), with a range of 5–84 alleles per cow (reads per allele ranged from 410 to 12,388; Figure 7A), excluding seven outlying cattle samples with significantly higher allele counts (C42—570 alleles, 6,399 reads; C40—298 alleles, 7,922 reads; C21—242 alleles, 7,994 reads; C45—210 alleles, 7,465 reads; C75—207 alleles, 9,828 reads; C26—204 alleles, 9,229 reads; C55—192 alleles, 7,708 reads). In contrast, the median allele count in buffalo was 484 (SD = 111.1), with a range of 298–684 alleles per buffalo (reads per allele 319–524; Figure 7A). When allele sharing within and between species was analyzed, 1,248 Tp4 alleles were unique to cattle (including TpM, #0001), 3,201 were unique to buffalo and no alleles were shared.

For Tp16, amplicons from 95 samples (73 cattle, 21 buffalo, and TpM) were sequenced (no duplicates, total *n* = 95). Sequence data were not generated for three cattle samples and after filtering, a further 12 cattle samples were lost, resulting in post-filtering data for 58 cattle samples, as well as all buffalo samples and TpM. A total of 4,215 alleles were initially identified, but after filtering (a sequence must be represented by two independent reads) the final allele count was 1,451 (175,395 reads). A single TpM allele was identified (3,948 reads) at the 1% SNP/INDEL variant frequency threshold used (Figure 6C). The median allele count in cattle samples was 3 (SD = 6.9), with a range of 1–9 alleles per cow (reads per allele 1–7,252; Figure 7B), excluding four outlying cattle samples with significantly higher reads (C25—42 alleles, 3,636 reads; C14—26 alleles, 1,868 reads; C60—24 alleles, 987 reads; C24—17 alleles, 6,139 reads). The median allele count in buffalo was 275.5 (SD = 80.8), with a range of 153–369 (reads per allele 438–1,822; Figure 7B), excluding one outlying buffalo sample with a significantly lower allele count (B20—3 alleles, 5,283 reads). Analysis of allele sharing identified 58 Tp16 alleles unique to cattle (including TpM, #0080), 1,389 alleles unique to buffalo, and four shared alleles (#0002 present in 16 cattle and 2 buffalo; #0014 in 12 cattle and 2 buffalo; #0016 in 2 cattle and 2 buffalo; #0052 in 1 cow and 3 buffalo).

**FIGURE 8:**
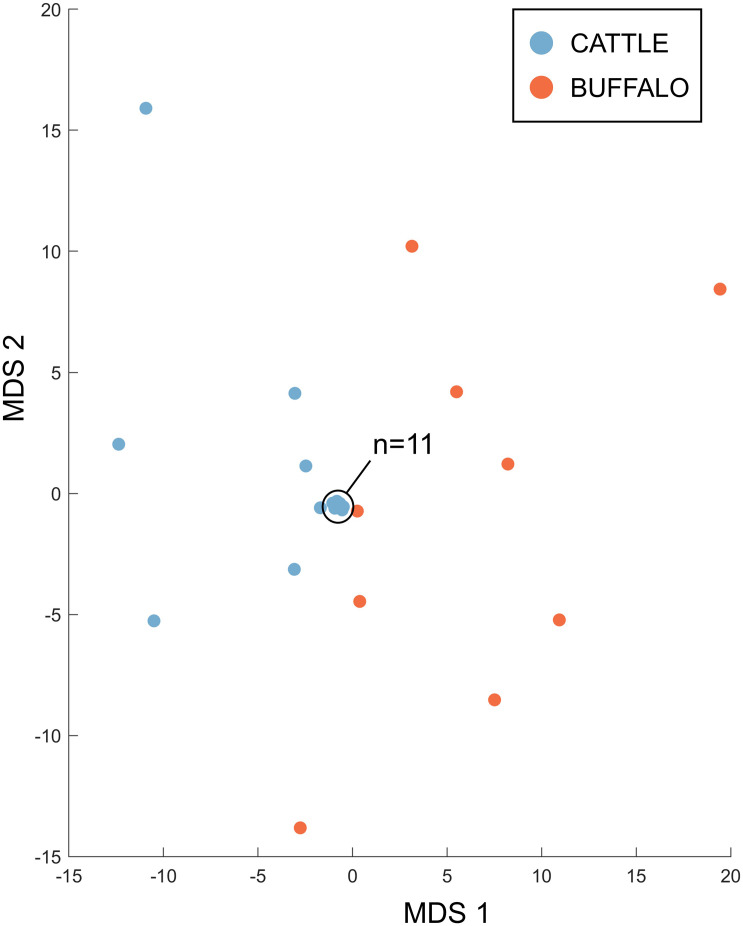
Multidimensional scaling analysis incorporating counts for non-singleton alleles from samples for which data was generated for all three antigens. Data across the three antigens were integrated; the first two dimensions are shown. Cattle samples (*n* = 18) are represented in blue and buffalo samples (*n* = 9) in orange.

Therefore, the Tp4 and Tp16 data showed the same trends as seen in Tp1, with significantly higher allele numbers observed in the buffalo samples, and limited evidence of sharing alleles between species. When data for all three antigens was integrated, for the 18 cattle and 9 buffalo samples that had data for all three antigens, it was evident that the cattle and buffalo samples represented largely non-overlapping populations (Figure 8), with greater distances between the buffalo samples, and the cattle samples mostly clustering very tightly together.

## Discussion

This study aimed to assess *T. parva* genetic and antigenic diversity at the population level, in sympatric cattle and buffalo, by analyzing the sequence of near full-length amplicons of *T. parva* antigen genes Tp1, and Tp4 and Tp16. Samples deriving from cattle and buffalo in the same time and space enable the analysis of key unresolved questions around the epidemiology of *T. parva*—to what degree are parasites shared between buffalo and cattle, and can this inform on disease risk as well as potential utility of tools such as vaccination? A risk in analyzing *T. parva* from buffalo-endemic areas is the circulation of closely related *Theileria* species, which can confound results through cross-amplification. Significant care was taken to ensure that the PCR primers designed did not cross-amplify with other species, and particularly with the closely related *T*. sp. (buffalo) (Bishop et al., 2015), and so we are confident that the amplicons are *T. parva-*specific. For Tp1, at 1% threshold, a high level of diversity at the nucleotide level was observed, with 651 allele variants being identified across the 46 samples (34 cattle, 12 buffalo), and importantly only a single allele in the control clonal TpM sample. There was greater allelic diversity seen in buffalo, with a median of 81.5 alleles (SD = 45.2) being identified in buffalo compared to a median of nine alleles (SD = 21.8) in cattle samples. Additionally, there was overall a higher number of alleles detected in buffalo (*n* = 420) than in cattle (*n* = 239), despite there being much fewer buffalo samples. Only a small number of alleles (*n* = 8) were found in both species. These data suggest that the method described provides an increased resolution for detecting *T. parva* antigen alleles over those in previous studies. The results are in agreement with the findings of several previous studies that there is greater *T. parva* parasite heterogeneity in buffalo-derived populations compared to cattle-derived populations (Bishop et al., 1994; Oura et al., 2011a; Pelle et al., 2011; Kerario et al., 2019). The Tp1 alleles in the study population showed a high degree of relatedness, consistent with the samples all deriving from a largely co-circulating population. While there was limited support for genetic substructuring by host, time or location, there was evidence that there may be some incipient divergence between parasites deriving from cattle and buffalo, with one large group of buffalo-derived samples clustering together and being rarely found in cattle—in contrast to a second cluster in which multiple of both cattle- and buffalo-derived alleles were found.

The analysis of Tp4 and Tp16 showed the same pattern of greater allelic diversity in buffalo compared to cattle, and with relatively few alleles shared between species. The difference in diversity was particularly marked for Tp16, which had very low allele counts in cattle-derived samples compared to buffalo (3 vs. 275 median number of alleles, respectively). This may reflect functional restriction on diversity in Tp16 when *T. parva* is in cattle compared to buffalo, but also may be due to the lower overall levels of polymorphism in Tp16, resulting in a cleaner signature of differential polymorphism between cattle- and buffalo-derived parasites.

Of note, when a SNP/INDEL variant frequency threshold of 1% was used for Tp4, there were thousands of alleles detected, including multiple alleles for the TpM control sample derived from a clonal laboratory line. However, on closer examination this was as a result of multiple INDELS, which were found to correlate with homopolymer runs (more frequent in Tp4 than in Tp1 or Tp16), and thus likely to derive from PacBio error, as homopolymer stretches are an acknowledged issue with PacBio (Weirather et al., 2017). The SNP/INDEL frequency threshold was adjusted to the level (5%) at which only a single TpM allele was detected (Figure 6B), but this threshold will likely result in a conservative estimate of allele numbers. Additionally, analysis of Tp4 was complicated by the presence of five introns. The allele counts reported above were calculated based on exonic sequence only. Notably, the INDEL and SNP incidence was significantly higher in the intronic regions (Figure 6A). This provides indirect validation of the PacBio approach and the analytical pipeline used, as higher mutation rate would be expected in non-coding sections of DNA not under obvious functional restriction. It also provides some indication of the background mutation rate in these populations, with the average number of insertions, deletions and SNPs being 37, 24, and 75 and 13, 12, and 35 in buffalo-derived and cattle-derived samples (introns, 5% threshold), respectively, further emphasizing the reduced diversity in the cattle *T. parva* samples.

As outlined above, multiple alleles were detected in every animal, indicating a high level of multiplicity of infection. Hemmink et al. (2018) had previously observed an average of 16 Tp1 alleles in individual buffalo from Kruger, South Africa, and 14 in Ol Pejeta, Kenya—a total of 72 alleles from 14 animals, also using an amplicon sequencing approach, albeit amplifying a smaller (344 bp) region of Tp1. The number of alleles identified in buffalo in our study was much higher (median of 81.5), which may reflect the increased amplicon size of 1,618 bp, but also suggests that the PacBio approach and analytical pipeline results in greater resolution in terms of allele detection. While PacBio can introduce errors into the sequencing data, we included TpM amplified from *in vitro* cells as a control, and with our analysis only one allele was detected in TpM, as expected, giving confidence to the alleles detected in the field samples. Mixed genotype infections of *T. parva* are common in field samples, and have been suggested to derive from parasite diversity within the tick vector population (Elisa et al., 2015), which can be amplified through sexual recombination in the tick (Katzer et al., 2011; Henson et al., 2012). However, ticks usually have only very few infected salivary gland acini, and the sporozoites in each acinus normally originate from a single parasite (Gitau et al., 2000), which in turn suggests that extensive allelic diversity within individual animals is likely to be the result of cumulative infections through multiple and sequential tick bites. This is supported by previous studies of cattle in Uganda using satellite DNA markers, which showed an increase in number of alleles with age (Oura et al., 2005).

The allelic polymorphism observed in Tp1 was predominantly composed of synonymous SNPs, but did include non-synonymous SNPs, including five variants observed in the previously defined epitope that is recognized by protective CTL responses against Tp1 (MacHugh et al., 2009; Connelley et al., 2011); the epitope variant in the reference stock, TpM, was VGYPKVKEEML, as expected (Graham et al., 2008; Pelle et al., 2011; Elisa et al., 2015; Hemmink et al., 2018). Epitope variant VGYPKVKEEII was most frequently observed in cattle (31) and VGYPKVKEEML was most common in buffalo (all 12). Several other studies have demonstrated that -ML is the dominant epitope across East Africa, being the predominant allele detected in cattle in Kenya, Tanzania, Burundi, Democratic Republic of Congo, Malawi, Rwanda and Uganda (Pelle et al., 2011; Elisa et al., 2015; Amzati et al., 2019; Kerario et al., 2019; Atuhaire et al., 2020; Chatanga et al., 2020; Nanteza et al., 2020). Pelle et al. (2011) is the most comparable study to ours, having examined Tp1 diversity in parasites isolated from buffalo or from cattle with varying exposure to buffalo-derived *T. parva*. That study found variant –ML in the majority of their isolates in Kenya (6 of 17 cattle-derived laboratory isolates, 16/27 cattle-derived field isolates, 22/25 buffalo-associated cattle isolates [(i.e., from cattle co-grazed with buffalo) and 13/16 buffalo-derived isolates]. –II, the next most common variant in Pelle’s dataset, suggested possible association with cattle (38% of cattle samples compared to 13% of buffalo-derived or associated samples), but our data indicated that –II was frequently found in both cattle and buffalo. Of the rare variants in the Pelle dataset (-IL in one cattle-derived cell line and -MI in one buffalo-associated cattle cell line), -IL was found in both cattle (6) and buffalo (1), and -MI was only found in one cow in our samples. Kerario et al. (2019) also examined Tp1 alleles in cattle associated with buffalo in five areas of Tanzania, and found variant –ML in the majority of their samples (48/98 cattle, 17/19 buffalo-associated cattle and 6/13 buffalo-derived), and similar to Pelle et al. (2011), found the next most common variant to be –II (38/98 cattle, 1/19 buffalo-associated cattle and 2/13 buffalo-derived samples). Although in our dataset epitope variant –MV was only observed in buffalo, it was recently reported in a single cow from Mara region in northern Tanzania—the cow having had no obvious history of co-grazing with buffalo (Kerario et al., 2019). Therefore, our data add to previous reports and agree that epitope -ML is common in *T. parva* cattle samples. In our study, epitope -II is the most frequent in cattle, but this is also commonly found in other populations, and without further work it is difficult to conclude if this is meaningful in terms of relating to epidemiological differences. We did detect all five known Tp1 epitopes in the sample set, suggesting that this is a genetically diverse *T. parva* population—probably reflecting the large resident population of both buffalo and cattle, and the lack of tick control impact on the tick population in protected areas. However, it may also reflect the improved resolution of the protocol employed, suggesting this may prove advantageous in future studies aiming to define *T. parva* genetic diversity.

While there was limited evidence for genetic substructuring in our data, and the presence of alleles shared between cattle and buffalo does indicate that there is (probably infrequent) mixing of parasite populations, it may be that the occasional transmission between cattle and buffalo, and genetic exchange in ticks, is sufficient to blur the signature of any genetic drift that may be happening between subpopulations. It may also simply reflect that the diverse *T. parva* population evolved in buffalo before the arrival of cattle in Africa, and that the cattle-maintained *T. parva* parasite population is a subset of this ancient buffalo-derived population. However, the indications of separate clusters of genotypes that derive from either only buffalo or from both cattle and buffalo, alternatively suggest that this may be an example of incipient speciation, with divergence between cattle and buffalo-derived samples in process, i.e., comparable to but at an earlier stage of divergence than the very closely related *T*. sp. (buffalo) (Palmateer et al., 2020)—albeit that in *T*. sp. (buffalo) this is suggested to be due to adaptation to a different tick species rather than a different mammalian host (Bishop et al., 2015).

There were a variety of potential factors that could have influenced potential substructuring of the *T. parva* population, and we did collect sample metadata that included host species, date of sampling, place of sampling and management practice. However, for each antigen the number of samples resulting in successful generation of sequence data were small for each category, meaning the data was under-powered for analysis of any genetic association. Although spatiotemporal analysis was not formally carried out, given the close relatedness of the sequences overall there was not an evident trend in allele distribution across space or time in the data.

It is well established that buffalo-derived *T. parva* do not differentiate well into the piroplasm state in cattle (Young et al., 1977; Sitt et al., 2015), and most attempts to transmit infection from cattle infected with buffalo-derived *T. parva* have been unsuccessful (reviewed in Morrison et al., 2020). Since the cattle samples in this study were almost all from healthy animals, the *T. parva* detected predominantly represent the carrier state cattle-maintained parasite population (i.e., *T. parva* that is able to differentiate to transmissible infections in cattle). The inability of buffalo parasites to undergo differentiation to the piroplasm stage in cattle is believed to result in a barrier to maintenance of buffalo-derived parasites in the cattle population, thus accounting for less genotypic diversity in the cattle *T. parva*. However, in a few instances experimental feeding of large numbers of ticks on cattle experimentally infected with buffalo parasites has resulted in low level transmission and further passage by ticks in cattle, yielding parasites that behave similarly to cattle *T. parva* (Barnett and Brocklesby, 1966; Young and Purnell, 1973; Maritim et al., 1992). Whether or not such adaptation occurs naturally at a sufficient level to allow the parasites to establish in cattle is unclear. Previous comparisons of the genotypes of buffalo and cattle parasites have focused mainly on cattle parasites from buffalo-free areas. Therefore, it was of interest in the current study, where cattle are exposed to buffalo parasites, to determine whether there is greater diversity in the cattle *T. parva* population, possibly representing introgression of buffalo *T. parva* genotypes into the cattle parasite population. However, the cattle parasite population detected showed less diversity compared to the buffalo population and the genotypes were largely different in the two host species, with most antigen alleles unique to one or other host species and only comparatively few shared. While there has been considerable focus on the transmission of buffalo-derived *T. parva* to cattle, there have been very few studies to examine infection of buffalo with cattle-derived *T. parva.* Our data suggest that, although very few alleles are directly shared, one clade of Tp1 alleles is found regularly in both cattle and buffalo, indicating that there is some degree of transmission between buffalo and cattle within this clade.

The level of interaction between cattle and buffalo, and the ticks that feed on them, in the study area is currently not quantifiable. The limited number of alleles found in both hosts likely reflects that acute infections of cattle with buffalo-derived parasites could not be examined in this study, and this highlights the need for further studies to examine parasites in clinical cases of *T. parva*, in order to assess the impact of buffalo-derived parasites upon clinical disease burden in this epidemiological setting. The national park boundary is unfenced, and buffalo (and ticks) are free to move into suitable habitat outside the national park, although fragmentation of habitat and increasing human population may mean that this is occurring less frequently. Discussion with farmers in the study area did reveal that grazing of cattle within the protected areas does occur (particularly seasonally when water supplies are limited) providing opportunity for ticks to feed on both cattle and buffalo. In order to fully understand the parasite dynamics across this wildlife-livestock interface, it would also therefore be necessary to examine the parasites circulating in the tick population, to establish the parasite genotypes they carry, as well as which hosts they are feeding on.

Only a relatively low proportion (7.87%) of the 1,602 cattle samples tested were positive for *T. parva*, and only some of these resulted in successful amplification of Tp1, Tp4, and Tp16 (likely due to the samples representing low parasitaemic carrier state, as well as the relative efficiency of the PCR assays). The limited number of sympatric buffalo samples reflected the difficulty in obtaining such samples. A further limitation of the current study is that by focusing on antigens recognized by the bovine immune system, we may be analyzing genes that are under particular selection pressures, which has the potential to confound other genetic signals, such as those associated with population substructuring by host. In order to more fully understand the population structure, ideally a larger sample set of both *T. parva-*positive cattle and buffalo would be required, and an approach such as sequence capture used to enable analysis of polymorphism at the whole genome level (Palmateer et al., 2020). Analysis of whole genome data would facilitate unbiased and thorough analysis of genetic diversity in cattle and buffalo-derived *T. parva*, and enable identification of signatures of selection that may underpin adaptation to differentiation to piroplasms in cattle, for example. A greater understanding of the pattern of such diversity across the geographic range of *T. parva*, in particular focusing on the remaining areas where cattle and buffalo interact, would be particularly enlightening in terms of the overall population structure, and potentially how much a role buffalo-derived *T. parva* play in disease epidemiology across the parasite’s range.

The shape of the parasite population in cattle and buffalo has important implications for use of the ITM vaccine. As has been observed previously, cattle vaccinated with ITM are unlikely to be protected against heavy challenge with buffalo-derived parasites (Sitt et al., 2015). Therefore, the increased allelic diversity in buffalo in our study site, and in particular the clade of Tp1 alleles only found in buffalo, presumably reflects a substantial pathogen diversity that cattle may be exposed to if fed on by infected ticks. How effective the ITM vaccine would be in this area may depend on the extent of exposure to buffalo-derived parasites, which as outlined above could not be captured with the current sample set. Additionally, frequent tick challenge resulting in exposure to multiple cattle-derived *T. parva* genotypes may be sufficient to confer broad protection prior to encountering buffalo-derived infection, and this also is an aspect that could not be inferred from our data.

*T. parva* is an interesting example where there has been a relatively recent species jump by the pathogen, and provides a prime case study for adaptation of a complex eukaryote pathogen to a new host. This study established and validated a novel genotyping pipeline to analyze genetic and antigenic diversity of *T. parva* by long read sequencing, allowing analysis of near full-length sequences from three polymorphic antigen genes, importantly in samples derived from sympatric cattle and African buffalo. These data shed light on the complex interplay between *T. parva* populations in buffalo and cattle, revealing the extent of the significant genetic diversity in the buffalo *T. parva* population, the limited sharing of parasite genotypes between the host species, and highlighting that a subpopulation of *T. parva* is maintained by transmission within cattle. The results emphasize the importance of obtaining a fuller understanding of buffalo *T. parva* population dynamics in particular, as ultimately a more holistic understanding of the population genetics of *T. parva* populations will enable a realistic assessment of the extent ofbuffalo-derived infection risk in cattle, and how this may impact upon current control measures such as vaccination.

## Data Availability Statement

Data presented in this study and scripts used to analyse the data are available via the following link: https:/doi.org/10.7488/ds/3003.

## Author Contributions

FA, SJ, WM, HA, and LM designed the study. FA, EP, ES, TK, RF, FM, ST, TL, JH, PT, and HA carried out the field work and generated the samples and data. FA, SJ, IH, HA, WM, and LM undertook analysis and interpretation. All authors wrote the manuscript.

## Conflict of Interest

The authors declare that the research was conducted in the absence of any commercial or financial relationships that could be construed as a potential conflict of interest.

## Footnotes

1 https://www.pacb.com/products-and-services/analytical-software/multiplexing/

2 https://github.com/PacificBiosciences/CCS

3 https://github.com/PacificBiosciences/barcoding

4 https://github.com/PacificBiosciences/blasr

5 https://doi.org/10.7488/ds/3003
